# Supplementary figures and images for: Production and provenance of architectural glass from the Umayyad period
Source: PLoS One. 2020 Sep 28;15(9):e0239732. doi: 10.1371/journal.pone.0239732 (PMC7521681; doi:10.1371/journal.pone.0239732)

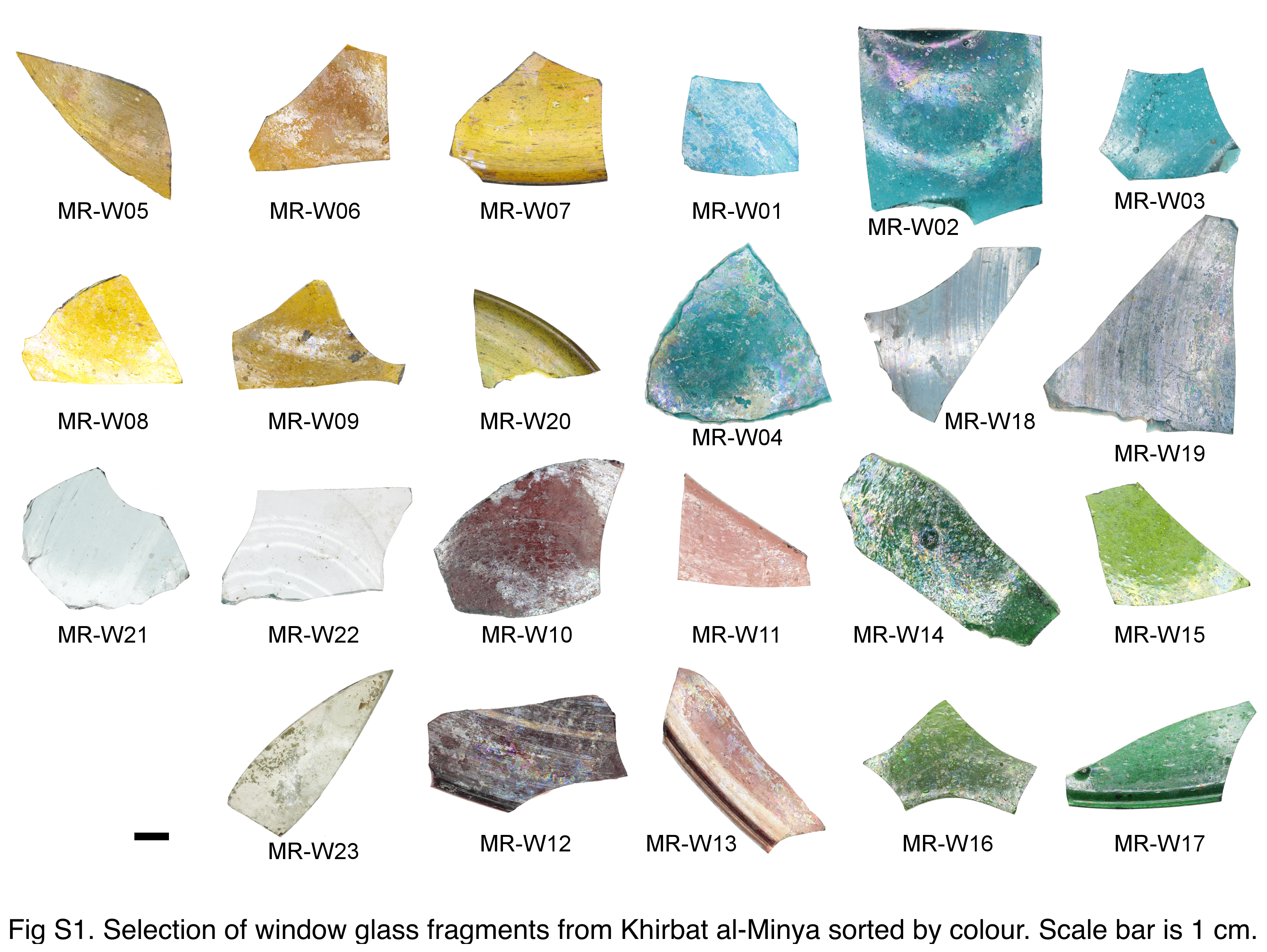

Supplement: S1 Fig — Scale bar is 1cm. (TIF) [file pone.0239732.s001.tif]
