# Supplementary material for: Production and provenance of architectural glass from the Umayyad period
Source: PLoS One. 2020 Sep 28;15(9):e0239732. doi: 10.1371/journal.pone.0239732 (PMC7521681; doi:10.1371/journal.pone.0239732)
Supplement: Attachment — Submitted filename: Adlington PLOS ONE.pdf [file pone.0239732.s004.pdf]

This study looks at chemical compositions by Laser Ablation ICP-MS of 270 tesserae and 37 window glass sherds from the Umayyad complex Khirbat al-Minya in Israel. A subset of 60 tess and 14 window glass were embedded in resin, polished and analyzed by EDS. The paper is well-written and one of the first studies looking at Umayyad tesserae. The results have important implications for understanding this transitional period in glass types and supplies; the implied dating of the complex from the dominance of Egyptian glass type, Egypt 1a, is intriguing and the same goes for the observed systematics for the colourant/opacifyers indicating that the secondary addition of these were done in a single workshop. The paper is appropriate for PLOS One once some critical points have been addressed.

1. The sample classification needs to be more detailed and include discussion of the possible presence of Egypt 1c.
2. There has to be a detailed section that look at recycling. There is no such discussion. Instead, it is ruled out based on one tesserae block found in the complex. It has to be dealt with – in particular the Levantine samples show clear indications of recycling and presence of pre-Apollonia glass such at that produced at Jalame.
3. Small, but important. There are not enough references to figures.

Author list:
What were author contributions? Since last author is corresponding author, is the order alphabetical?

Introduction:
Tesserae are found 1. as loose and embedded in mosaics with mortar bedding and 2. without mortar in a large deposit. '**Additionally, a fragment of a glass slab in the thickness of the tesserae was found with marks from impact …….., suggesting this was the material from which the tesserae were cut on site. This indicate that some, if not all, of the glass are contemporary to the site**.' This is a critical statement to the paper since they use it to conclude that all Egypt 1 and Levantine glass is primary and contemporaneous with the complex. Based on this, they conclude that the complex was build in AD 705-715 rather than 743-744. - Yet there is no description of the glass slab:
What was the glass type?
What was the colour?
Why are they convinced that the glass is contemporary to the site?
Why could it not be recycled translucent glass that was melted, coloured and shaped into the slab for tesserae production? If so, it could be older glass. There needs to be a discussion on

recycling rather than just dismissing it on the basis of one glass slab.

The last paragraph of the introduction states the purposes or rather the main observations (?) of the study, but is very vague. If the authors want to summarize their observations in the introduction, they should be specific.
Purpose or observation:

1. '**Using chemical compositions of glass from Khirbat al-Minya for an in-depth examination of the acquisition and circulation of glass during early 8th century'**.
   Bold statement when the paper deals with one site only. Be specific – what was learned about the glass acquisition and circulation?
2. "**Multiple mosaic colours appear to have been made at the same secondary workshop, yielding insights into the production model for secondary glass working'**.
   Again, grand statement – but be specific. What insights did it yield?

Methods:
A total of 270 glass tesserae and 37 window glasses were examined. Of there, 210 tess and 23 window glasses were analyzed by LA-ICPMS without sample prep (NS), while 60 tess and 14 window glasses (MR) were mounted in epoxy, polished and studied by LA-ICPMS, scanning electron microscope SEM-EDS. Why not do some repeats of the same sample by both methods for comparison to test the differences?

The authors use the Corning B, C and D standards for calibration, but then also for reproducibility/accuracy determinations. That does not make sense. The point of running external standards as unknowns is to choose some that would fall elsewhere on the calibrations curves – the same as unknown samples. I would strongly encourage that they only report the standards that were not used for calibration (Corning A and NIST SRM 612).

Line 109. Give some idea of the obtained reproducibilities and accuracies are. Just a couple of lines saying that the reproducibility was generally x % for elements above x ppm and x % for elements below x %. And do the same for accuracy.

Results:

Egypt1a - line 146-151 only 5 lines
The authors state that **'one third of tesserae and half of the window glass samples classify as Egypt 1a dated to first quarter of the 8th century'**. They base this on $Al_2O_3/SiO_2 > 0.05$,

TiO/Al2O3 = 0.07 and 70-120 ppm Zr (line 151) and refer to Fig. 1A. Yes, figure 1A implies that these are Egypt 1 type glasses, but not that they belong to subgroup Egypt 1a. The authors need to present arguments for why these samples are Egypt 1a (e.g. Sr/Ca, Zr/Th, La/TiO2 etc. Schibilles own work 2019). In fact, samples MR-T100, 103, 107 and 108 have Sr/Ca ratios around 0.013, which relate them to Egypt 1c and not Egypt1a according to Schibille et al., Fig. 1b. This should be addressed in the text.

Egypt1a sandsource - line 151-158

Line 151. '**3 samples, …., are similar to Egypt 1a in their sand-related elements including REE**'. Please elaborate on this statement and why not show this in a figure?

Is it even possible to use REE and sand-related elements or ratios to say these coloured samples have the same sandsource as Egypt 1a? Or is it more correct to say they have the same source as Egypt1?

Are REE patterns and other sand-related elements for Egypt 1a very characteristic compared to other natron groups and even other Egypt1 groups? From figure 2 in Schibille et al., 2019, it looks like Egypt 1a,b,c have very similar REE patterns. Since the authors are not using reduced compositions, it would be hard to differentiate between REE patterns for Egypt 1a,b,c since it looks like the ratios between the REE are similar.

Levantine - line 170-175 only 5 lines

The authors state that **half of tesserae and five window glass samples classify as Levantine with TiO2/Al2O3 < 0.05**. Figure 1A should be referenced here and not just in the beginning of the result section. They go on to classify the Levantine samples according to their Na2O/SiO2 ratios and CaO/Al2O3 ratios (Phelps et al., 2016) and conclude that they are Levatine 1 type glasses produced at Apollonia. I disagree that it is this simple (see examples below).

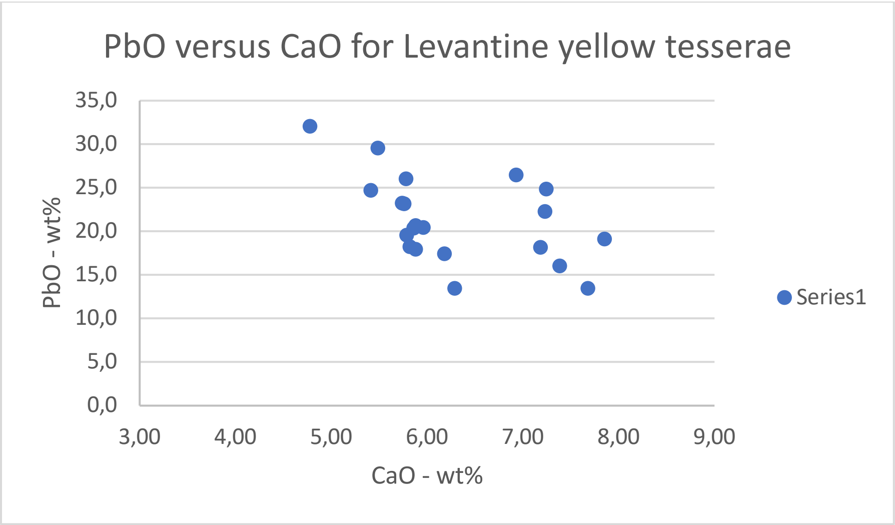

Above is a plot that shows PbO versus CaO for the yellow tesserae of Levantine type. Here, it looks like Pb-tin colourant/opacifyer was mixed with two distinct types of Lavantine base glass characterized by different CaO concentrations. If the samples are sorted according to CaO concentrations, it is clear that the samples with high CaO also have MnO concentrations above 0.02.

Sample	Base Glass	Colour	Na_2O	MgO	Al_2O_3	SiO_2	P_2O_5	Cl	K_2O	CaO	Ca	TiO_2	MnO	Fe_2O_3	SnO_2	PbO
[illegible]T127	Levantine	Yellow	8,23	0,35	2,24	47,0	0,04	0,52	0,34	4,78		0,05	0,01	0,32	3,64	32,0
[illegible]T011	Levantine	Yellow	10,2	0,48	2,21	51,9	0,05	0,66	0,39	5,41		0,06	0,02	0,33	3,14	24,7
MR-T121	Levantine	Yellow	9,66	0,44	2,06	47,0	0,05	0,59	0,32	5,49		0,05	0,02	0,31	4,09	29,5
MR-T129	Levantine	Yellow	10,2	0,44	2,47	52,1	0,04	0,61	0,35	5,74		0,06	0,01	0,34	4,00	23,2
MR-T132	Levantine	Yellow	11,0	0,48	2,32	51,7	0,07	0,58	0,49	5,76		0,06	0,02	0,36	3,62	23,2
NS-T009	Levantine	Yellow	9,25	0,44	2,35	50,6	0,05	0,58	0,44	5,78		0,06	0,02	0,37	3,48	26,0
NS-T013	Levantine	Yellow	10,2	0,51	2,36	57,2	0,06	0,68	0,42	5,79		0,06	0,03	0,36	2,56	19,5
MR-T131	Levantine	Yellow	10,2	0,45	2,42	58,1	0,05	0,70	0,38	5,82		0,06	0,02	0,34	3,08	18,2
MR-T122	Levantine	Yellow	10,4	0,47	2,43	55,6	0,06	0,65	0,49	5,86		0,06	0,02	0,33	2,97	20,4
MR-T133	Levantine	Yellow	11,2	0,45	2,44	54,2	0,07	0,64	0,38	5,88		0,06	0,02	0,38	3,29	20,6
MR-T135	Levantine	Yellow	10,4	0,46	2,40	58,3	0,06	0,72	0,39	5,88		0,06	0,02	0,34	2,98	17,9
MR-T130	Levantine	Yellow	11,1	0,48	2,46	54,8	0,05	0,64	0,40	5,96		0,06	0,02	0,35	2,81	20,4
MR-T125	Levantine	Yellow	11,5	0,55	2,27	57,8	0,13	0,69	0,55	6,18		0,06	0,03	0,34	2,13	17,4
MR-T137	Levantine	Yellow	10,3	0,51	2,61	62,3	0,05	0,71	0,47	6,29		0,07	0,02	0,38	2,63	13,4
MR-T136	Levantine	Yellow	10,3	0,51	2,20	48,0	0,16	0,54	0,87	6,93		0,06	0,35	0,40	3,08	26,4
MR-T134	Levantine	Yellow	11,7	0,59	2,29	54,6	0,13	0,84	0,66	7,18		0,06	0,30	0,42	2,89	18,2
MR-T138	Levantine	Yellow	10,2	0,47	2,46	52,6	0,09	0,64	0,52	7,23		0,06	0,02	0,38	2,48	22,3
MR-T124	Levantine	Yellow	9,81	0,54	2,34	50,0		0,62	0,77	7,25		0,06	0,42	0,44	2,22	24,9
NS-T012	Levantine	Yellow	12,3	0,64	2,35	56,9	0,11	0,79	0,63	7,38		0,06	0,22	0,40	2,03	16,0
MR-T128	Levantine	Yellow	12,8	0,64	2,49	59,0	0,12	0,80	0,63	7,68		0,07	0,27	0,44	1,50	13,4
MR-T139	Levantine	Yellow	11,0	0,51	2,51	53,8	0,11	0,64	0,57	7,86		0,06	0,05	0,40	2,37	19,1

Thus, while group 1 with low CaO could be from Apollonia, this cannot be the case for group 2 since base glass from Apollonia does not contain MnO. It is more likely a component of older Levantine glass from Jalame in the high CaO group.

The paper doesn't consider the possibility of recycling, but looking solely at the yellow tesserae, there is a correlation between K and P that is independent of Pb (and thus Pb-tin addition). See two plots for reported composition and reduced compositions. This indicates that these glasses are made of **recycled** Levantine baseglass contaminated by K and P during the remelting/recycling.

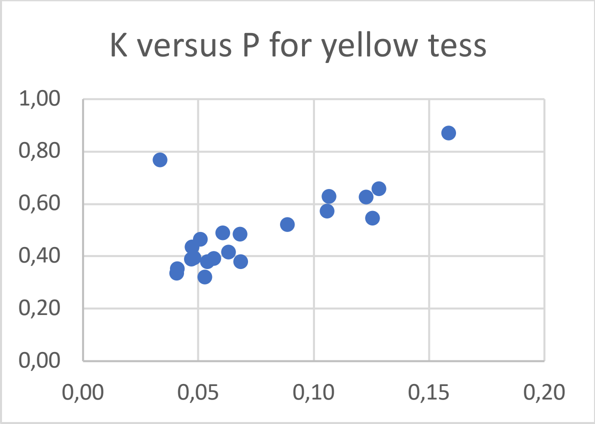

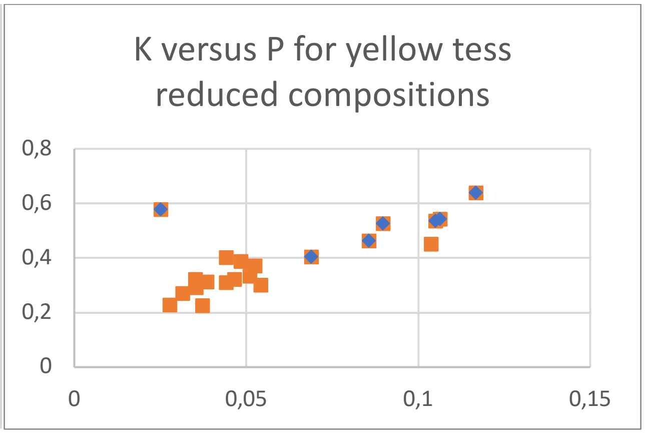

Similar considerations should be done for the other coloured groups. For the individual groups, the Ca/Al2O3 ratio (Phelps et al., 2016) is higher in samples with high MnO (or MnO/SiO2) – again, a sign that there is older byzantine (Jalame) glass present in the Levantine baseglass.

Colouring and opacifying

This is great, well-written section. I miss a comparison of the gold-leaf tesserae to the observations by Neri et al., 2016 (Glass and gold) – eventhough this paper is referenced, it is not discussed and the materials not compared.

Again for the gold leaf tesserae, it looks like there is a significant recycling component (see K-P correlation below) – and there could therefore be significant pre-Apollonia material in the Levantine gold leaf tess, but Mn cannot be used to fingerprint this since it is used for colourant in these.

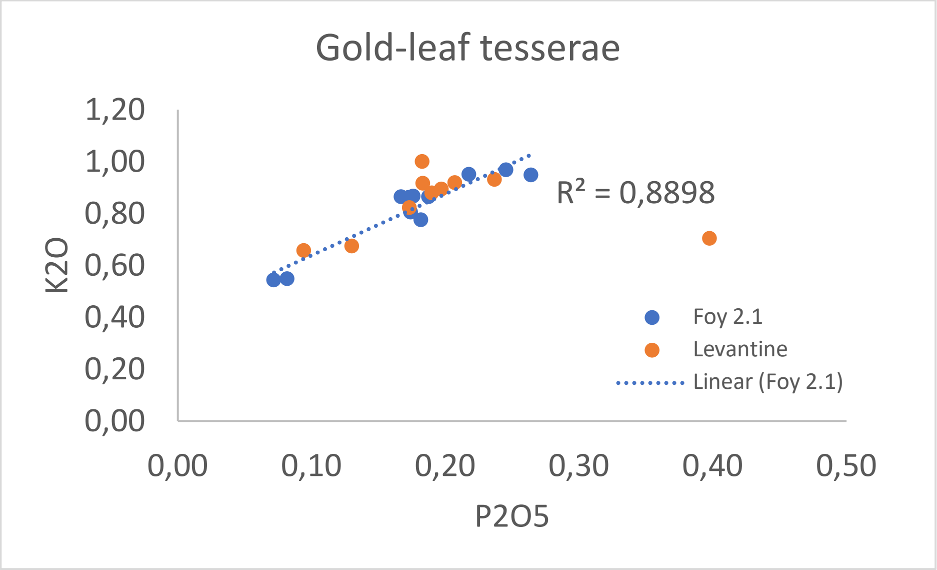

Line 266. 'The copper used to colour the Levantine tesserae have variable concentrations of zinc and tin'. Could this also be a reflection of the recycling?

Discussion

Line 382. Note that the Levantine glass used for gold leaf tesserae also could be older and reused (see above).

Line 392. Figure 4 is a critical figure in the text and should therefore be described and introduced in details. 'Figure 4 presents …..' rather than referred to once and then not again in the next paragraph although this is discussing the figure.

Conclusion

Line 433-434. 'Red, cobalt blue and gold leaf are made from primary Levantine glass'. Again, the authors need to address the recycled nature of the Levantine glass.

Minor comments:

Line 117. **'No permits were required for the described study, which complied with all relevant regulations'**. This sentence is out of context and belongs with sample selection.

Line 126. Typing error '**Tabe'** = Table

Line 163. '**CaO/Al2O3 versus Na2O/SiO2 contents'** should be changed to CaO/Al2O3 versus Na2O/SiO2 ratios.

Line 171. Levantine samples with TiO2/Al2O < 0.05 – refer to figure 1A here.

Line 230. Bi & Pb correlation for Egypt 1 samples. Refer to plot in figure 4.

Line 268. Provide a reference for gunmetal composition.

Line 274. Correlation CaO and P2O5 showing bone ash, again refer to plot in Figure 4 where this is shown.

Line 279. '**The gold leaf tesserae (Levantine)**', I think it should be The gold leaf tesserae (Levantine & Foy 2.1).

Line 322. Copper also underlies the green glasses. Confusing sentence.

Figures:

Figure 1 is very smudged so it is hard to see the details.
