# Supplementary material for: Production and provenance of architectural glass from the Umayyad period
Source: PLoS One. 2020 Sep 28;15(9):e0239732. doi: 10.1371/journal.pone.0239732 (PMC7521681; doi:10.1371/journal.pone.0239732)
Supplement: Attachment — Submitted filename: Response to reviewers2.docx [file pone.0239732.s006.docx]

**PONE-D-20_02692R1**

Dear Dr Henderson:

Thank you for the positive feedback on our revised manuscript *Production and provenance of architectural glass from the Umayyad period*. We have now extended our discussion of the two publications by Fiorentino et al that deal with similar material from Khirbat al-Mafjar, particularly as regards the production model of strongly colored mosaic tesserae. We thus hope that this answers to the request of a more detailed discussion of these two publications. We would like to draw your attention to the fact that the more refined early Islamic groups of Egypt 1a, 1b and 1c were published after the said articles and the discussion of the base glass types in Fiorentino *et al.*, 2018 is not up to date. Our interpretation is thus based on a re-evaluation of their compositional data (see figure below for your information). Similarly, some of their observations about secondary production models of workshops specifying in the production of a single color, and a possible diversification of suppliers in late antiquity have become obsolete, because of the very limited number of samples upon which they rest their assessment. Here, we propose a revised secondary production model using detailed evaluations of coloring and opacifying raw materials based on a statistically more robust number of samples.

Please find below also a version of the manuscript with track changes to facilitate the identification of the modifications.

I hope that our manuscript is now suitable for publication in PloS One.

Yours sincerely,

Laura Ware Adlington (for the authors)

**Editor Remarks to Author:**

… to refer to Fiorentino et al’s publications on similar material in AAS 2018/2019 more extensively and in much more detail.

We have expanded and clarified the base glass diversity (lines 436-439) and added a paragraph on the secondary production model based on the colouring and opacifying materials (lines 487-507), proposing a different scenario for the organization of secondary workshops dedicated to the manufacture of mosaic tesserae.
